# Trunnionosis in total hip arthroplasty with accompanying amyloidosis and calcium pyrophosphate deposition disease

**DOI:** 10.1016/j.radcr.2023.10.002

**Published:** 2023-10-31

**Authors:** Kevin Christopher Serdysnki, Sam Kaplan, Aaron Oraham, Sidney Mirgati, Fatima Akili, Emad Allam

**Affiliations:** a Loyola University Medical Center and Loyola University Chicago, 2160 S 1st Ave, Maywood, IL 60153 USA; b Rowan-Virtua School of Osteopathic Medicine, 42 E Laurel Rd, Stratford, NJ 08084 USA

**Keywords:** Trunnionosis, Metallosis, Adverse reaction to metal debris, Hip arthroplasty, Amyloidosis, Calcium pyrophosphate deposition disease

## Abstract

Metal-on-polyethylene (MoP) is a common type of total hip arthroplasty. Trunnionosis is a rare but concerning complication of this type of implant. Trunnionosis involves wear and corrosion at the femoral component's head-neck interface and may cause a local inflammatory response referred to as an adverse reaction to metal debris (ARMD). We present a rare case of trunnionosis with concurrent calcium pyrophosphate deposition disease (CPPD) and amyloidosis in a MoP implant. ARMD can be linked to crystalline arthropathy and amyloidosis, possibly due to metal ion-induced acidosis. Clinicians should consider trunnionosis in patients with persistent post-surgical hip pain, and order metal ion tests and synovial fluid analysis to aid in the diagnosis of this condition if suspected. Timely recognition enables appropriate surgical revision.

## Background

Nearly half a million hip replacements are performed in the United States annually. The modern system used for total hip arthroplasty (THA) often consists of 4 separate modular components: a femoral stem, femoral head, liner, and acetabular cup. In the past, a metal-on-metal (MoM) design was favored because a metallic cup and femoral head allow for greater stability, less wear, and theoretically lower rates of hardware failure. However, over time, MoM implants have been shown to wear at the cup-head interface and release metallic particles into the surrounding joint space. This causes adverse reaction to metal debris (ARMD), which is an umbrella term for soft tissue damage from metal particles. It is estimated that 20% of MoM implants will require revision surgery within 10-13 years [1].

MoM implants have largely been replaced by a metal-on-polyethylene (MoP) design, which has less than a 4% revision rate after 10 years [1]. However, metallosis and ARMD have also been identified in MoP implants. Unlike their MoM counterpart, MoP implants tend to wear at the head-neck interface of the femoral component; this region is called the trunnion. This process is known as mechanically assisted crevice corrosion or trunnionosis and is estimated to account for 3% of revision procedures [1,2]. Diagnosis is typically indicated by abnormally elevated serum cobalt or chromium levels. Previous reports have identified concomitant disease processes occurring with metallosis in MoM implants, including 3 cases of concurrent crystalline deposition disease, one of which also had concurrent amyloidosis [3,4]. We present a unique case of trunnionosis in a MoP implant with accompanying calcium pyrophosphate and amyloid deposition disease. This is the first reported case of these 3 coexisting conditions in a MoP implant.

## Case presentation

A 79-year-old male residing at a skilled nursing facility was admitted to the hospital for altered mental status, abdominal pain, and a recent fall. He had a history of bilateral MoP THA, in addition to hypertension, hyperlipidemia, hypothyroidism, coronary artery disease, and aortic stenosis. His surgical history included coronary artery bypass grafting, aortic valve replacement, and total colectomy with ileostomy placement. The left THA was performed for a proximal femoral fracture approximately 20 years ago. He reported occasional left hip/groin pain.

**Fig. 1:**
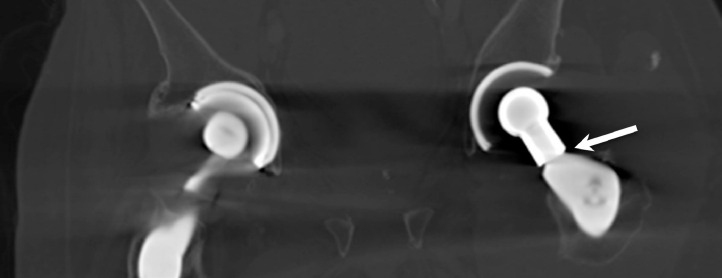
Coronal CT image of the pelvis with bone windows demonstrates modular design of the left total hip arthroplasty, with the femoral head-neck interface or trunnion indicated by the arrow.

**Fig. 2:**
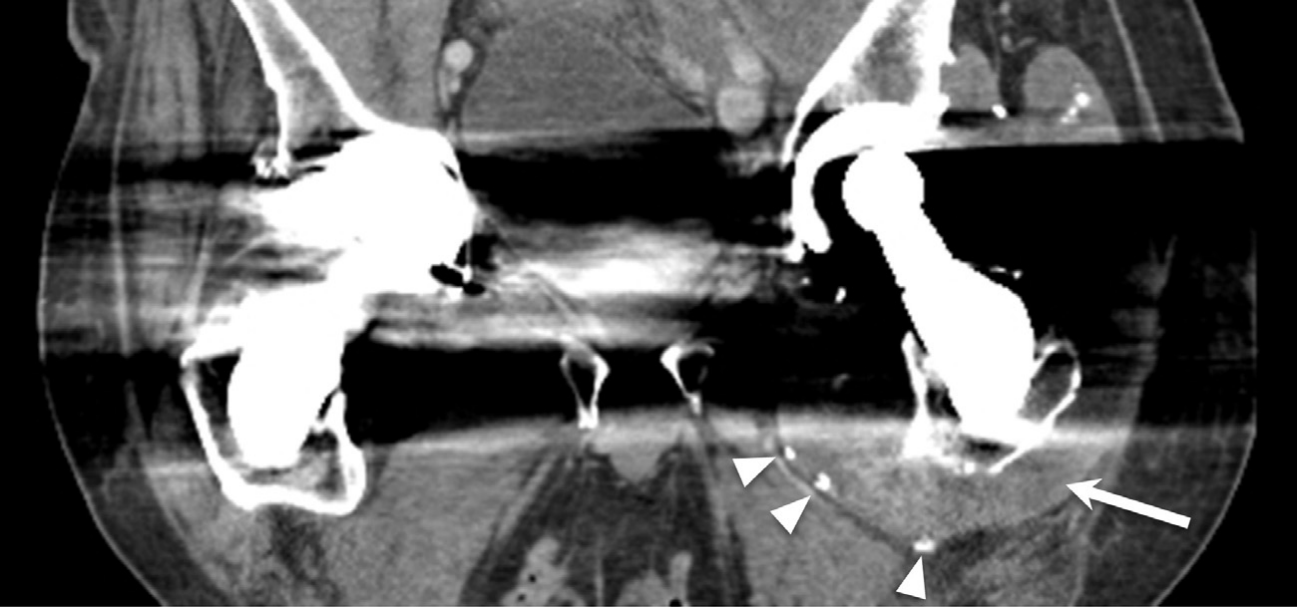
Coronal CT image of the pelvis with soft tissue windows demonstrates abnormal soft tissue attenuation around the left total hip arthroplasty (arrow). Evaluation is limited by streak artifact from the hardware. There are foci of metallic debris around the left hip (arrowheads).

**Fig. 3:**
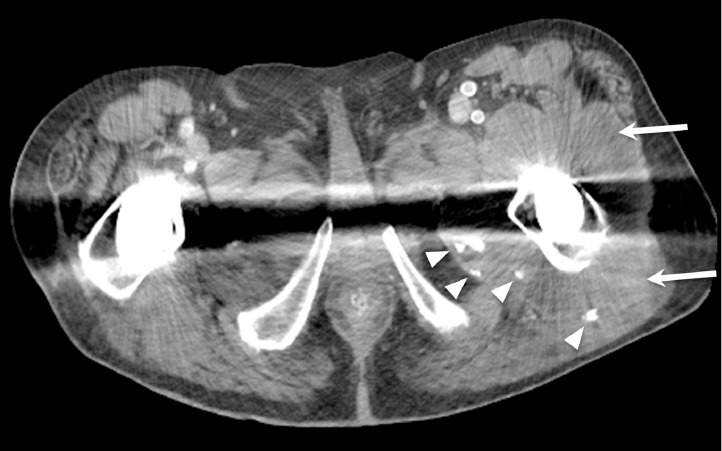
Axial CT image of the pelvis demonstrates pseudotumor formation (arrows) and multiple foci of hyperdense metallic debris (arrowheads) surrounding the left hip.

**Fig. 4:**
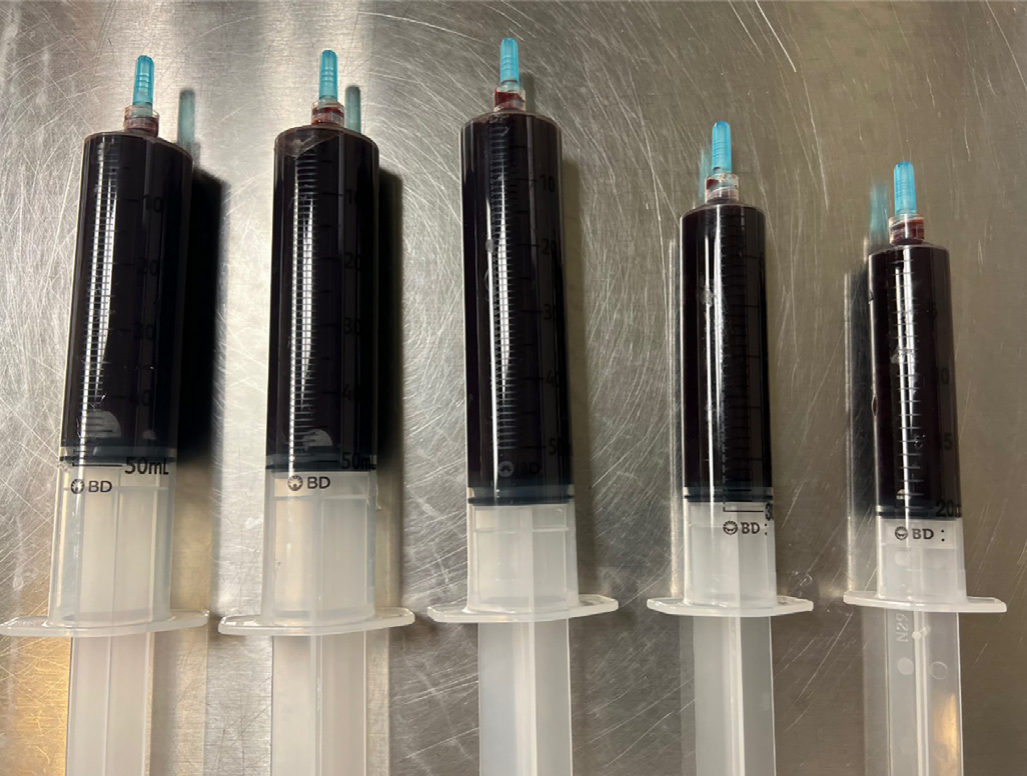
Ultrasound-guided left hip joint aspiration yielded a large amount of dense black fluid, highly indicative of metallosis.

CT demonstrated a fluid collection along the patient's left iliopsoas bursa and left hip with foci of metallic debris (Fig. 1, Fig. 2–3). Subsequently, the patient underwent ultrasound-guided needle aspiration of the left hip, and approximately 200 mL of black tarry fluid was obtained (Fig. 4). The aspirated fluid was positive for chromium (10.4 ng/mL) and calcium pyrophosphate crystals. Congo red stain was positive for amyloid. Bacterial cultures were negative. Empiric antibiotics were discontinued. The patient's hospital course was complicated by delirium. The patient was given haloperidol and trazodone with improvement in mentation. He actively participated in physical therapy and occupational therapy sessions during his hospital stay. The patient was discharged from the hospital to a skilled nursing facility. Plans for surgical revision of the left THA were deferred due to the patient's comorbidities.

## Discussion

The trunnion is a metal-on-metal interface at the femoral head-neck junction that can wear and cause ARMD in MoP implants. Although the exact pathophysiology is unclear, trunnionosis is likely caused by a combination of fretting, chemical corrosion, and metallic ion release, resulting in a lymphocyte-driven inflammatory cascade. Several manifestations of ARMD or ALTR (adverse local tissue reaction) have been reported: soft tissue necrosis, synovitis, aseptic lymphocyte-dominant vasculitis-associated lesion (ALVAL), and pseudotumor formation [5], [6], [7]. The most common symptoms and physical examination findings are nonspecific: pain, stiffness, weakness, and/or instability of the affected hip joint. Systemic symptoms are rare, but vision changes and neurological symptoms have been reported in a few cases [8]. Diagnosis is established through appropriate imaging, blood levels of cobalt and chromium, and synovial fluid aspiration.

Crystalline arthropathy typically presents as sudden onset joint pain and swelling with overlying erythema. The pathophysiology involves monosodium urate (gout) or calcium pyrophosphate (pseudogout) crystal deposition in the articular and periarticular space, leading to inflammation. In the case of pseudogout, also known as calcium pyrophosphate deposition disease (CPPD), the diagnosis is indicated by identifying rhomboid-shaped crystals with weakly positive birefringence in the synovial joint aspirate. Some important risk factors include advanced age, obesity, alcohol consumption, osteoarthritis, acidosis, recent joint surgery, and diabetes mellitus [9], [10], [11]. There have been 3 documented cases where ARMD was associated with crystal deposits, confirmed by synovial fluid analysis [3,4].

Amyloidosis is a broad term that describes the deposition of misfolded proteins in soft tissue, leading to inflammation and organ dysfunction. It is most commonly associated with multiple myeloma and autoimmune diseases, notably rheumatoid arthritis. Amyloid is identified under polarized light after staining with Congo red. There has been one documented case of ARMD in a MoM implant associated with amyloid deposition, confirmed by Congo red staining [3].

It remains unclear whether ARMD causes crystalline arthropathy and amyloidosis, or whether they are involved in the pathophysiology of ARMD. One possible explanation is metal ion-induced acidosis in the local soft tissues. Acidosis is a known risk factor for crystalline arthropathy and is suspected to contribute to protein misfolding in amyloidosis [3,4]. Here we reported a case of trunnionosis and ARMD in a patient with a MoP hip implant with accompanying amyloid and CPPD confirmed by synovial fluid analysis.

## Conclusion

Trunnionosis is a complication of MoP hip replacement surgery that can result in pain and hardware failure and may be associated with concomitant joint disease. Clinicians should include trunnionosis in the differential diagnosis for patients with MoP implants complaining of hip pain, especially if there is evidence of ARMD on imaging. In addition to checking for metal ions, clinicians should check for concomitant amyloid and crystal deposition when a joint aspiration is performed. The prevalence of amyloid and crystal deposition in ARMD is largely unknown due to inadequate screening. When identified, neither process should be treated in isolation. Definitive treatment involves surgical revision of the failed implant.

## Patient consent

Informed consent for this case was obtained from the patient.
